# Accuracy of computer‐assisted drilling of equine cervical vertebral bodies using a purpose‐built cervical frame—An experimental cadaveric study

**DOI:** 10.1111/vsu.14271

**Published:** 2025-05-15

**Authors:** Thimo Maurer, Mathieu de Preux, Christina Precht, Beatriz Vidondo, Christoph Koch

**Affiliations:** ^1^ Division of Equine Surgery, Swiss Institute of Equine Medicine (ISME), Department of Clinical Veterinary Medicine Vetsuisse‐Faculty, University of Bern Bern Switzerland; ^2^ Division of Clinical Radiology, Department of Clinical Veterinary Medicine Vetsuisse‐Faculty, University of Bern Bern Switzerland; ^3^ Veterinary Institute for Public Health, Vetsuisse‐Faculty, University of Bern Bern Switzerland

## Abstract

**Objective:**

To assess the accuracy of computer‐assisted surgery (CAS) of equine cervical vertebrae using a purpose‐built cervical frame (CF) for neck stabilization.

**Study design:**

Experimental cadaveric study.

**Sample population:**

Six whole fresh equine cadavers.

**Methods:**

Cadavers were positioned in dorsal recumbency with the neck extended within the CF. A cone‐beam computed tomography (CBCT)‐based surgical navigation system with optical tracking was used. A ventral approach exposed cervical vertebrae C3–C5. In each cadaver, 12 drill corridors were prepared with the patient tracker on the CF (position CF), followed by 12 corridors with the patient tracker on C3 (position C3). Surgical accuracy aberration (SAA) was assessed by measuring Euclidean distances between planned and executed entry and target points on merged pre‐ and postoperative datasets. Descriptive statistics and repeated‐measures analyses of variance (rep.‐meas. ANOVA) compared SAA measurements between groups.

**Results:**

The mean ± SD SAA (Euclidean distance) was 2.00 ± 0.98 mm in patient tracker position CF, and 2.41 ± 1.31 mm in position C3 (rep.‐meas. ANOVA *p* = .215). At the most dorsal point of the drill corridor, dorsoventral deviations >2 mm occurred in 5/72 measurements in patient tracker position CF, and in 12/72 measurements in position C3.

**Conclusion:**

The CF allowed for unrestricted pre‐ and intraoperative CBCT imaging and computer‐assisted drilling with a SAA in the close range of 2 mm. Positioning the patient tracker on the CF, outside the surgical field, did not compromise surgical accuracy.

**Clinical significance:**

A CF can facilitate CAS for surgeries with a ventral approach to the equine cervical vertebral column.

## 1 INTRODUCTION

Cervical vertebral compressive myelopathy is a common cause of spinal ataxia in horses and presumably the most frequent indication for surgery involving the cervical vertebral column in horses.^1^, ^2^ The general surgical treatment concept is vertebral interbody fusion through a ventral approach.^1^, ^2^, ^3^ This can be achieved with a basket implant bridging two adjacent cervical vertebrae.^2^, ^3^ Alternatively, locking compression plates^4^ and more recently polyaxial screw‐ and rod constructs^5^ or a three‐dimensional (3D)‐printed titanium cervical spacer and plate system^6^ have been used. Irrespective of the implant used, the demands on surgical precision in the immediate proximity of the vertebral canal are high.

Computer‐assisted surgery (CAS) can improve the precision and safety of implant insertion compared with conventional intraoperative image guidance.^7^, ^8^, ^9^ CAS mainly relies on computed tomography (CT) imaging coupled with navigation systems using optical tracking. Throughout a navigated procedure, the surgical navigation system continuously displays the location of the tracked surgical instruments on the patient's virtual anatomy on preoperatively acquired 3D imaging data. This provides the surgeon with real‐time 3D anatomic orientation and instrument guidance and allows for minimally invasive approaches while omitting repeated intraoperative image acquisition. Because no repeated imaging is necessary during the surgery, radiation exposure to personnel is reduced.

In equine orthopedic surgery, CAS using optical tracking systems has proven useful for various indications.^10^, ^11^, ^12^ Although this technology carries great potential to facilitate surgical interventions involving the equine cervical vertebral column, it has not been introduced for this application. In small animal surgery, the feasibility and safety of using CAS for placing implants in the thoracolumbar spine of dogs has recently been assessed in a cadaveric study.^13^ This trend follows what has become established in human spinal surgery, where 3D navigation systems are routinely applied.^14^, ^15^

In CAS with optical tracking, a patient tracker establishes the spatial relationship between the patient anatomy and the virtual, CT‐image‐based anatomy. The main prerequisites for accurate CAS are a stable fixation of the patient tracker with the targeted anatomy and an unrestricted direct line of sight between the detector camera and the tracked instruments during the navigated procedure. Typically, this is achieved by securing the patient tracker to the target anatomy near the surgical field. However, anchoring the patient tracker to a rigid frame remote from the surgical field is strategically advantageous, as it minimizes interference between the patient tracker and the tracked instruments while ensuring unrestricted access to the surgical field. Furthermore, complications associated with drilling pins to anchor the patient tracker can be avoided. This concept has already been applied in equine surgery, where a purpose‐built frame that stabilizes the distal extremity is used to facilitate CAS.^10^, ^16^

Considering the limited access and deep surgical approach to the ventral cervical vertebral column in horses, an external frame that allows patient tracker positioning remotely from the surgical field would facilitate CAS of this area. Moreover, for ventral interbody fusion surgery in horses, stabilization of the cervical vertebral column is essential to ensure proper alignment of the targeted vertebrae. This requires meticulous positioning of the horse and involves special padding and/or custom‐made troughs.^2^ Thus, a functional external frame for CAS of the cervical vertebral column in horses would have to integrate the requirements for cervical vertebral alignment, provide rigid fixation of the cervical vertebrae during the entire procedure, and minimize artifacts during cone‐beam CT (CBCT) imaging.

This study aimed to develop and evaluate the utility of a purpose‐built cervical frame (CF), designed to stabilize and align the cervical vertebrae for ventral interbody fusion surgery using CAS. The CF was intended to provide the necessary stability for CAS, while positioning the patient tracker outside the surgical field. Its utility was evaluated by assessing surgical accuracy aberrations (SAA) of computer‐assisted drilling procedures on whole fresh equine cadavers using two different patient tracker positions: one where the tracker was anchored to the CF and another where it was attached to one of the targeted cervical vertebrae. We hypothesized that the CF would enable unrestricted pre‐ and intraoperative CBCT imaging and accurate computer‐assisted drilling in both patient tracker positions. We also hypothesized that surgical accuracy would be highest in the vertebra closest to the patient tracker and decrease in more distant vertebrae.

## 2 MATERIALS AND METHODS

### 2.1 Cervical frame

**FIGURE 1:**
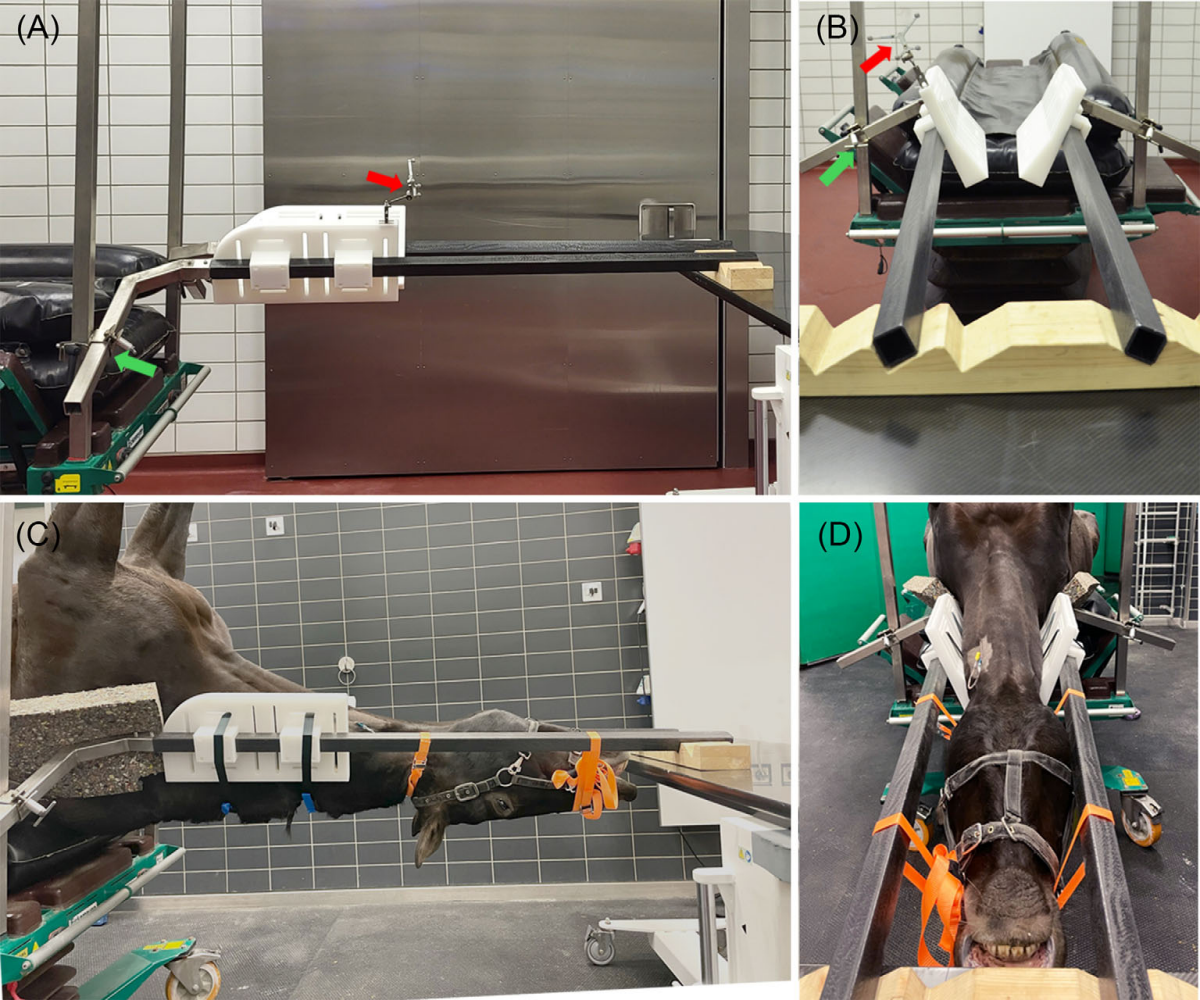
Cervical frame and cadaver positioning. (A, B) The core element of the cervical frame is a dual carbon rail system with two lateral panels to stabilize the horse's neck. The carbon rails are joined to a bent metal profile, which allows attachment to the operating table using custom‐made clamps (green arrow). Milled‐in recesses allow for rigid attachment of the patient tracker (red arrow). (C, D) Whole equine cadaver in dorsal recumbency, with the extended neck positioned within the cervical frame.

A CF was designed to allow for stable fixation of the cervical vertebral column in nearly horizontal alignment, with the equine cadaver positioned in dorsal recumbency (Figure 1A,B). The core element of this CF is a dual epoxy carbon rail system with two lateral panels to stabilize the horse's neck. Each rail is joined to a metal profile, which is readily attached to a commercially available operating table (Telgte II Surgery, Haico Oy, Finland). The design allows for patient‐specific height and distance adjustment between the carbon rails. The rails are stabilized by a wooden block resting on a height‐adjustable table near the horse's head. The two lateral panels are made from a hard plastic polymer (polyoxymethylene). The panels are positioned on the carbon rails as needed to accommodate the patient's size and anatomy, optimizing the stabilization of the cervical vertebrae. A gap between the panels, allows the crest of the neck to pass through so that the neck will not twist and so that the transverse processes of the cervical vertebrae become wedged between the panels. Milled‐in recesses allow for rigid attachment of the patient tracker to the panels in the most advantageous position. Furthermore, slots to pass straps were incorporated into the panels to enhance neck stability and optimize positioning.

### 2.2 Cadaveric specimen

Fresh cadavers of horses euthanized for reasons unrelated to this study and without any known history of cervical spine pathology were used. Appropriate methods of euthanasia, according to AVMA guidelines, were followed. Cadavers were donated after owners had signed an informed consent permitting the use of tissues and images for research purposes.

### 2.3 Preparation of cadavers

Within 2 h of euthanasia, cadavers were placed in dorsal recumbency, with the neck straightened and aligned in a neutral to extended position within the CF (Figure 1C,D). Wedge‐shaped foam cushions were inserted between the CF and the neck to fill gaps and help stabilize the neck. A standard ventral surgical approach^2^ was made to access the vertebral bodies of C3–C5. The ventral surface of the vertebral bodies was dissected free from the longus colli muscle using a periosteal elevator. A 10 mm radiopaque spherical head screw (Unibody Bone Fiducial; Medtronic, Louisville, Colorado) was anchored on the caudal aspect of the ventral crest of C3, C4, and C5 to serve as fiducial markers during the experimental procedure. The operating table and the height‐adjustable table near the horse's head, along with the cadaver secured in the CF, were then simultaneously adjusted in height to allow positioning of a mobile CBCT (O‐arm O2 Imaging System; Medtronic) unit's open gantry for imaging. Following preoperative imaging, the position of the cadaver and CF remained unchanged throughout the experiments.

### 2.4 Study design

**FIGURE 2:**
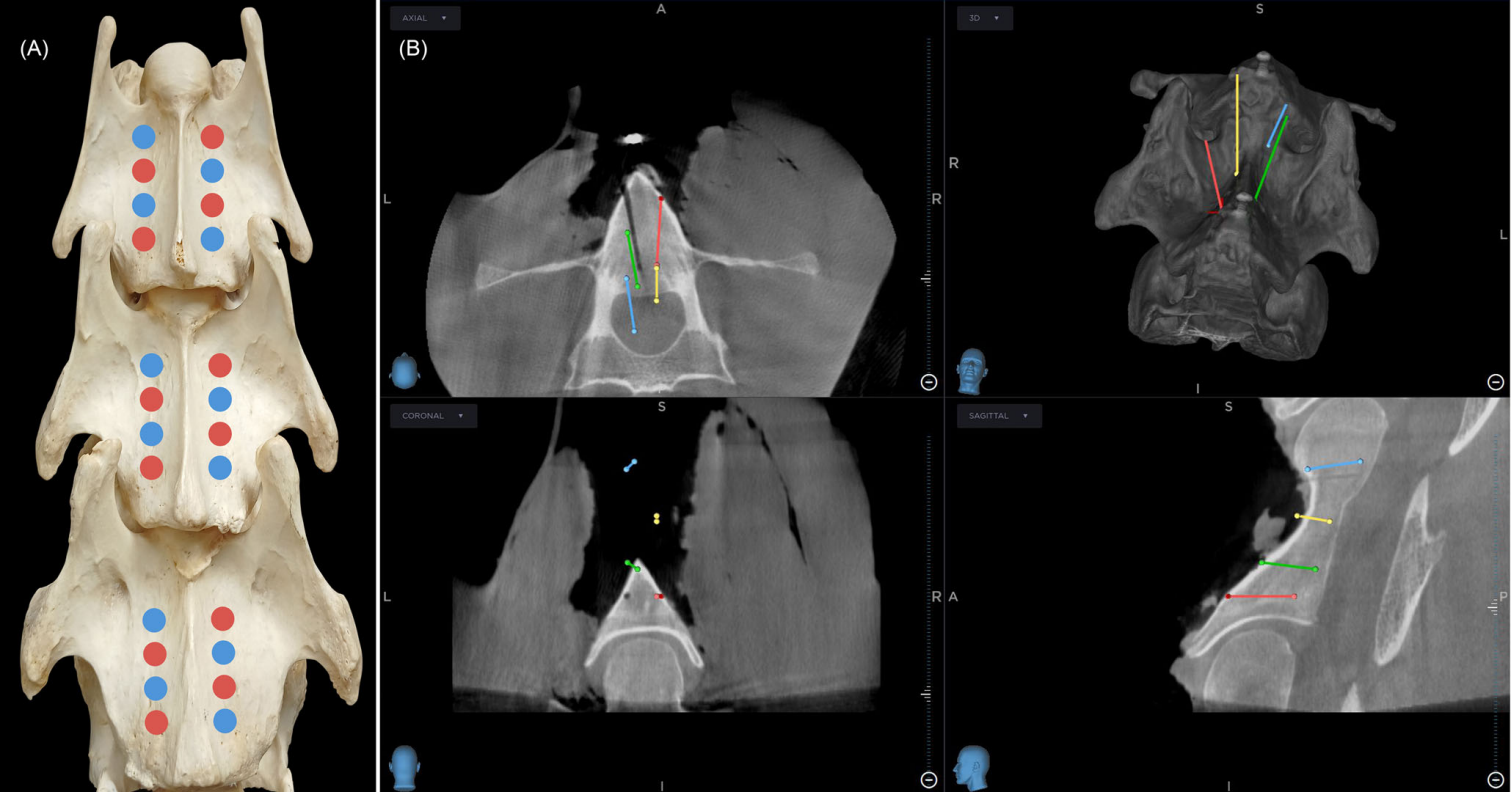
Study design and surgical planning. (A) In each cadaver, computer‐assisted drilling was performed on three cervical vertebrae (C3–C5) using two different patient tracker positions, illustrated here with blue and red dots. In each patient tracker position, four drill corridors were drilled per vertebra, alternatingly on the left and right body side. The drill corridors in the second patient tracker position were planned to mirror the first corridors. (B) Screenshot of the navigation monitor during surgical planning, showing a multiplanar reconstruction and a 3D model of a cone beam computer tomographic scan of a cervical vertebra and four surgical plans. The entry and target points of the anticipated drill corridors were set on transverse (top left), dorsal (bottom left) and sagittal (bottom right) images. The colored lines connecting the entry and target points represent the central axes of the planned drill corridors. The multiplanar reconstruction is centered on the entry point of the red surgical plan, which is mirrored to the existing drill corridor on the opposite side. Other surgical plans are displayed as overlays on this reconstruction.

The same procedural steps, consisting of preoperative image acquisition, surgical planning, computer‐assisted drilling, and postoperative image acquisition, were applied in 6 cadavers, and in each cadaver with two different patient tracker positions. A patient tracker (passive orthopedic reference frame 9730605, StealthStation System, Medtronic) equipped with infrared reflective spheres was firmly attached to milled‐in depressions of the CF's lateral panel on the left body side (position CF) or directly on the ventral crest of C3 (position C3) using the same spinous process clamp (Double Spinous Process Clamp Short, model 9734724, Medtronic). The order of the patient tracker positioning was determined with a coin toss for cadavers 1, 3, and 5, and reversed in each subsequent cadaver. In both patient tracker positions, four corridors were drilled on C3, C4, and C5 (in this order), alternatingly on the left and right body side (2 per side), resulting in 12 drill corridors per patient tracker position and cadaver (Figure 2A). After completing the navigated drillings using the first patient tracker position, the patient tracker was changed to the second position, and all procedural steps were repeated. The 12 drill corridors in the second patient tracker position were planned to mirror the first 12 drill corridors on the midline of the vertebral body. All navigated drilling procedures were performed by the same investigator (CK), experienced in computer‐assisted surgical procedures, navigated drilling, and equine spinal surgery. This investigator was positioned on the horse's right side for all surgical procedures. Spatial interference with the patient tracker and whether this required adjustment of the surgical plan or repeated image acquisition was recorded whenever encountered. The muscular stiffness of the neck caused by rigor mortis was scored from 0 to 2 (0 = no stiffness; 1 = mild stiffness; 2 = stiff) after all procedures on a cadaver had been performed.

### 2.5 Pre‐ and postoperative image acquisition

Preoperative CBCT images were acquired with a mobile CBCT unit and a surgical navigation system using optical tracking (StealthStation S8; Medtronic). Regardless of patient tracker position, C3, C4, and C5 were imaged in three subsequent scans (exposure of 120 kV and 80 mA, cylindrical volumetric data of 21 × 16 cm, and a voxel size of 0.415 × 0.415 × 0.833 mm^3^). The gantry position was saved at each vertebral level using the memory function of the mobile CBCT unit's control panel. During each CBCT scan, the localizer camera of the StealthStation S8 simultaneously detected the reflecting spheres of the patient tracker and the infrared light‐emitting tracker of the CBCT gantry. CBCT images were then automatically transferred to the surgical navigation system. After preoperative image acquisition, the position of the wheels of the mobile CBCT unit was marked on the floor with adhesive tape, and it was moved away from the surgical field.

Postoperative CBCT images of C3, C4, and C5 were acquired immediately after the 12 drill corridors had been prepared in a given patient tracker position. The mobile CBCT unit was moved into the same position as for the preoperative scans, using the markings on the floor for orientation, and the gantry was positioned consecutively over each of the three vertebral levels using the position memory function of the mobile CBCT unit. The same settings were used for the pre‐ and postoperative CBCT scans.

### 2.6 Surgical planning

Drill corridors were planned in the vertebral body of C3, C4, and C5 using the Cranial Software (Synergy Cranial Software, Medtronic) by one investigator (TM). The entry and target points were set on transverse, dorsal, and sagittal images (Figure 2B). Surgical planning was carried out on each vertebra immediately before computer‐assisted drilling of the same vertebra.

### 2.7 Computer‐assisted drilling

**FIGURE 3:**
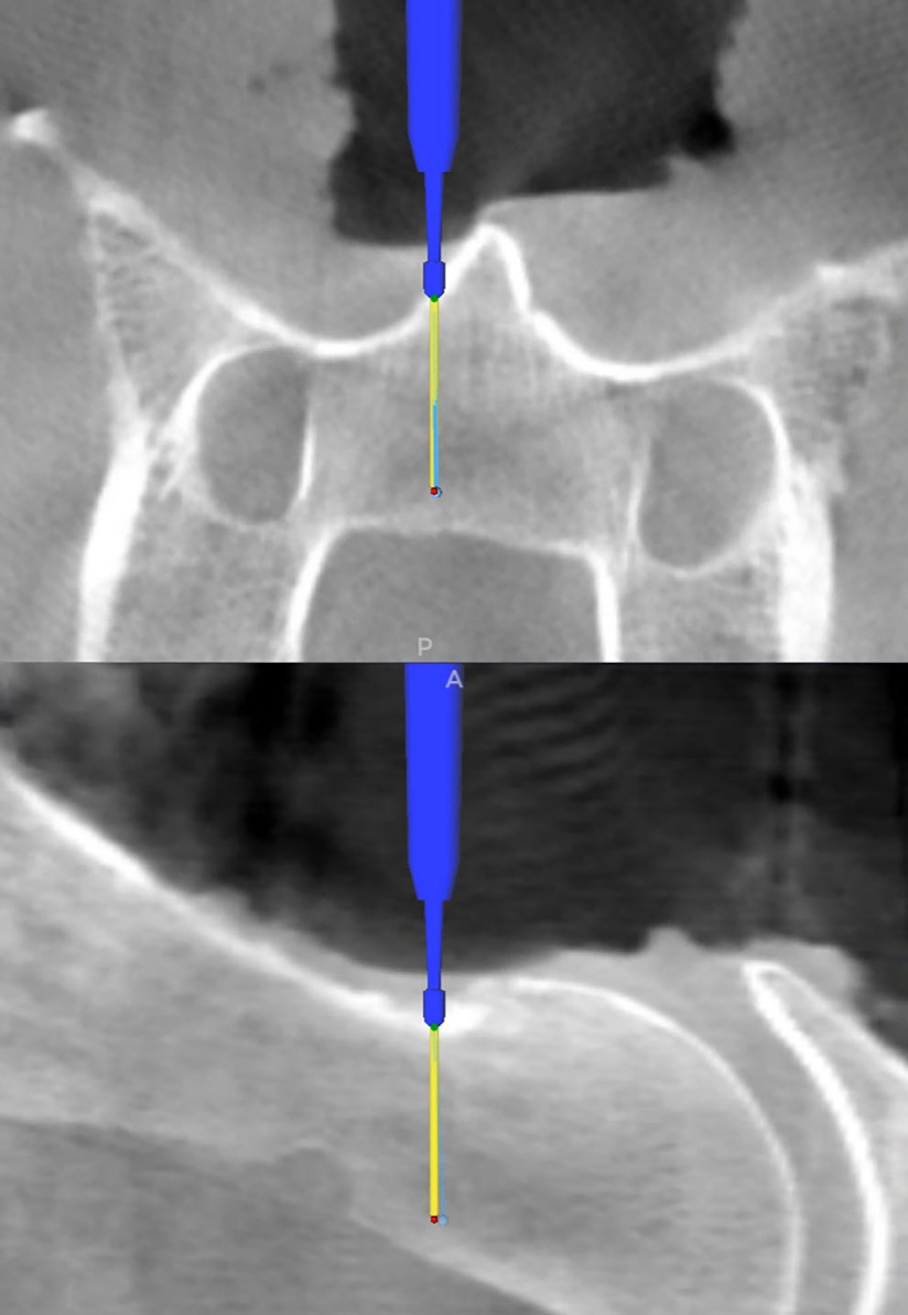
Computer‐assisted drilling. Screenshot of trajectories 1 and 2 as displayed on the navigation monitor during computer‐assisted drilling. The virtual tip of the high‐speed drill is shown at the entry point of the surgical plan on the vertebral surface. The yellow line represents the instrument tip's projection, guiding the surgeon to align the drill with the surgical plan (light blue line) before initiating drilling.

**FIGURE 4:**
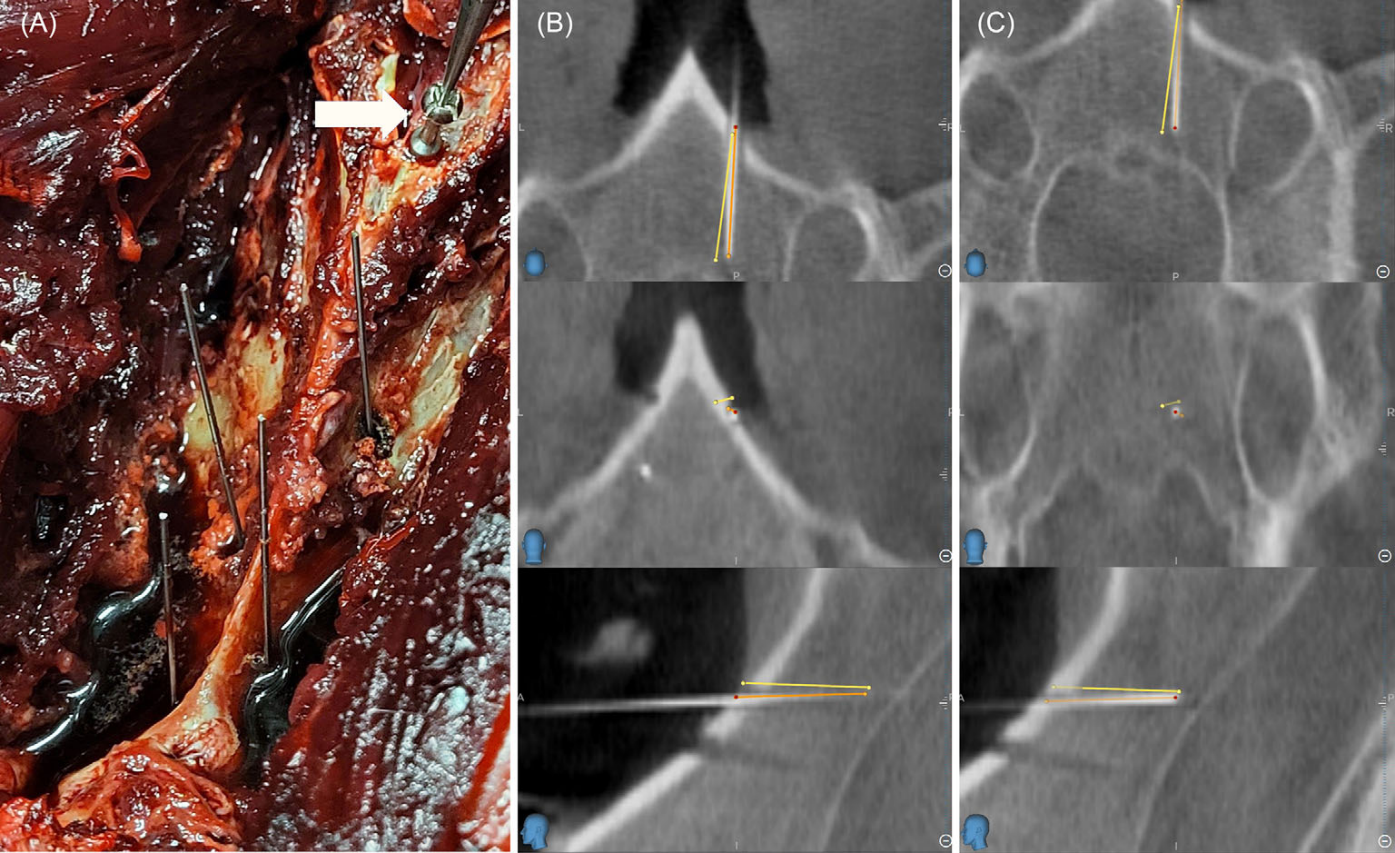
Surgical accuracy assessment. (A) Four titanium rods inserted into the drill corridors. Also note the spherical head screw (white arrow) anchored to the caudal aspect of the vertebra's ventral crest and the tip of the navigated pointer put into its central pit to assess the set‐up stability. (B, C) Multiplanar reconstruction of merged pre‐ and postoperative cone beam CT images shown on the navigation monitor. Annotations were manually set at the center of the achieved entry (B) and target (C) points of the drill corridor, easily identifiable by the radiopaque titanium rod, on transverse (top), dorsal (middle), and sagittal (bottom) plane images. The yellow line represents the surgical plan, while the orange line indicates the central axis of the drill corridor. To illustrate, a drill corridor with a rather large surgical accuracy aberration of 2.5 mm at the entry point and 2.3 mm at the target point was selected for clarity.

When surgical planning was completed, patient registration was performed by contacting one of the divots of the patient tracker with the tip of the navigated pointer (Passive Planar Probe [sharp], 960–553, StealthStation System, Medtronic). The Stealth‐Midas MR8 high‐speed drill (Medtronic) equipped with a small‐bore attachment (MR8‐10, Medtronic) and a preregistered 2.2 mm match head tool (MR8‐15MH22, Medtronic) was used for computer‐assisted drilling. The navigation mode was then selected, and trajectories 1 and 2 and the guidance function were displayed on the screen of the StealthStation S8. The navigated instrument was used to identify the entry point of the surgical plans. A virtual extension of the drilling tool was used to facilitate the alignment of the drill with the surgical plan on the screen (Figure 3). Throughout computer‐assisted drilling, the alignment of the drill with the surgical plan and its penetration depth were continuously monitored on the screen of the surgical navigation system. Drilling was discontinued once the tip of the drill reached the target point of the surgical plan. A 0.88 mm diameter titanium rod was inserted into the drill corridor to facilitate identification of each drill corridor on the postoperative CBCT images (Figure 4A).

### 2.8 Assessment of the set‐up stability

The stability of the whole set‐up was repeatedly verified by positioning the navigated pointer in the central pit of the spherical head screw placed on the corresponding vertebra before and after computer‐assisted drilling of the four drill corridors per patient tracker position and vertebra. A significant loss of set‐up stability was defined as present if the pointer tip would no longer be displayed being in contact with the virtual image of the screw head when placed in the core of the spherical head screw. This ensured the detection of a loss in accuracy exceeding 2.5 mm in any dimension.^16^ CBCT image acquisition and patient registration were repeated in case of significant loss of set‐up stability.

### 2.9 Surgical accuracy assessment

To assess the surgical accuracy of computer‐assisted drilling, the corresponding pre‐ and postoperative CBCT‐datasets of each vertebra were merged using the StealthMerge function of the Cranial Software (both Medtronic). Annotations were manually centered on the achieved entry and target points of the drill corridor (Figure 4B,C). Patient coordinates, including each surgical plan's entry and target point, and the annotations were exported from the navigation system. The Euclidean distance between the intended and achieved entry and target points was calculated using the formula: error = √ [(*x* ^2^ − *x* ^1^)^2^ + (*y* ^2^ − *y* ^1^)^2^ + (*z* ^2^ − *z* ^1^)^2^], where *x* ^1^, *y* ^1^, and *z* ^1^ represent the coordinates of the planned entry (or target) point, and *x* ^2^, *y* ^2^ and *z* ^2^ the coordinates of the achieved entry (or target) point, and defined the SAA in mm. In addition, at each entry and target point, deviations in the lateromedial (transverse plane/x‐axis), in the dorsoventral (sagittal plane/y‐axis), and in the caudocranial (dorsal plane/z‐axis) direction were assessed individually.

### 2.10 Statistical analysis

The sample size was estimated using R (version 4.2.2; R Core Team 2022), based on a previous study using the same CAS equipment and a similar study design.^16^ The minimum sample size required to detect an effect size of 0.6 mm with SD 1.17 is 24 drill corridors per vertebra and patient tracker position to achieve a power of 80% with a significance level of 0.05 (24 drill corridors in both patient tracker positions, totaling 48 drill corridors). Hence, we included six equine cadavers to prepare four drill corridors per patient tracker position and vertebra. Collected data were analyzed in NCSS 2024 Statistical Software (2024; NCSS, LLC. Kaysville, Utah). Descriptive statistics were performed on the Euclidean distance and axis‐specific SAA measurements (x‐, y‐ and z‐axis deviations in mm) in the two patient tracker positions. In addition, we report the number of y‐axis SAA measurements that exceeded 2 mm at the target point in both patient tracker positions. Normality of the outcome variable distribution was tested using Shapiro–Wilk and Kolmogorov–Smirnov tests, and logarithmic transformation was used where required to achieve normality. The effect of patient tracker position (CF vs. C3), vertebral level (C3, C4, C5), side (left vs. right), analysis point (entry or target point), and drilling chronology (first vs. second patient tracker position within each cadaver) on SAA was determined using a mixed regression model (repeated‐measures analysis of variance [rep.‐meas. ANOVA]) with the horse as the subject variable. The significance level was set at *α* = 0.05.

## 3 RESULTS

Six whole fresh equine cadavers were used for this study. Breeds included four Warmbloods, one Franches‐Montagnes, and one Thoroughbred. Two were females, and four were geldings. Ages ranged from 5 to 28 years, and the bodyweight from 389 to 603 kg.

The set‐up stability remained consistent in all experiments, meaning that the navigated pointer showed contact with the spherical head screw on patient images when placed in its central pit. Spatial interference between the instrument and patient tracker was documented on five occasions, all occurring at the C3 vertebra in patient tracker position C3. This required adjustments to the surgical plan on three occasions. At completion of the procedures, five cadavers showed a stiffness score of 2, while one cadaver had a stiffness score of 0.

**FIGURE 5:**
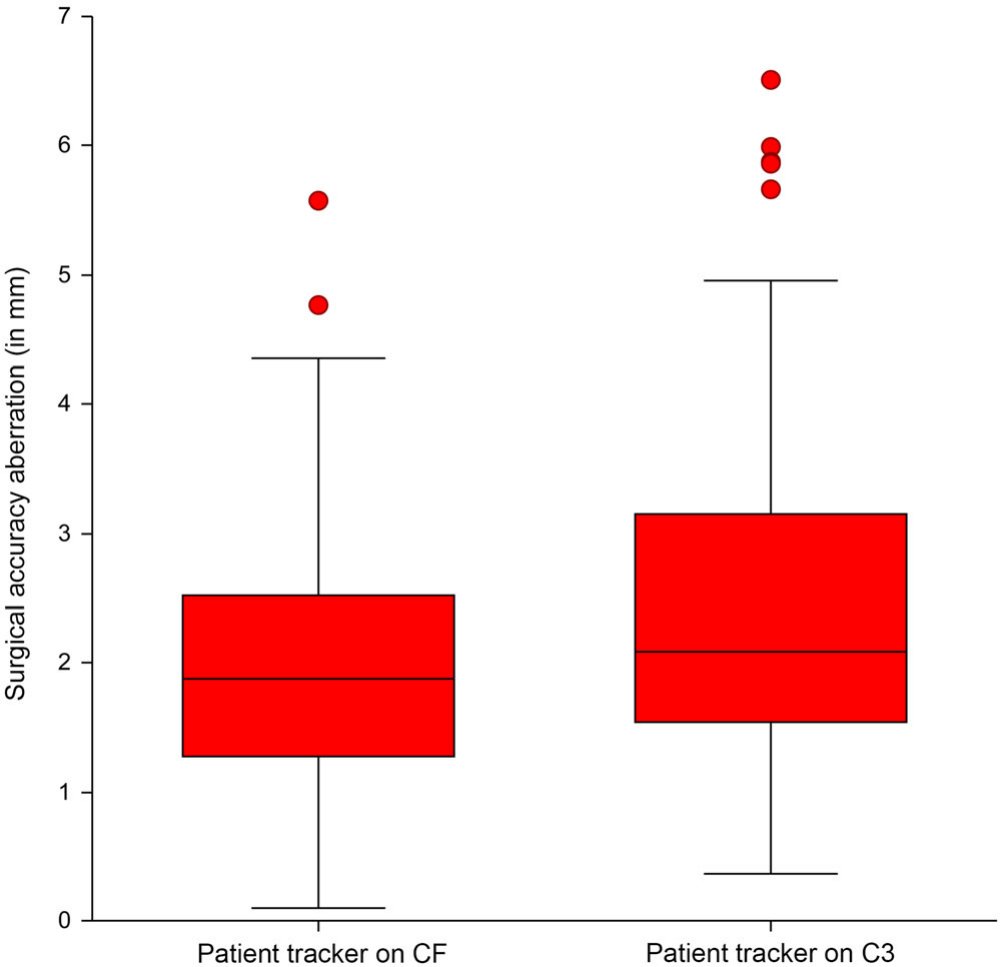
Box plot showing the overall surgical accuracy aberration (Euclidean distance) measurements in the two different patient tracker positions. CF, cervical frame, C3; third cervical vertebra.

The overall SAA (Euclidean distance) and axis‐specific SAA were not normally distributed, so the log‐transformed values were used. The mean ± SD SAA (Euclidean distance) was 2.00 mm ± 0.98 mm in patient tracker position CF, and 2.41 mm ± 1.31 mm in position C3 (rep.‐meas. ANOVA *p* = .215, Figure 5 and Table 1). Comparing SAA measurements between different vertebral levels, the mean ± SD SAA was 1.94 mm ± 1.04 mm on vertebrae C3, 2.17 mm ± 1.13 mm on vertebrae C4 and 2.51 mm ± 1.28 mm on vertebrae C5 (Table S1); rep.‐meas. ANOVA (*p* = .10). Neither analysis point (*p* = .23) nor side (*p* = .70) nor drilling chronology (*p* = .32) showed evidence of influencing the achieved SAA.

**TABLE 1:** Distribution of surgical accuracy aberration (SAA) in mm (Euclidean distance).

SAA	Overall (entry + target)		Entry point		Target point	
Patient tracker position	CF	C3	CF	C3	CF	C3
Mean	2.00	2.41	2.00	2.33	2.00	2.50
SD	0.98	1.31	1.01	1.33	0.96	1.29
Median	1.88	2.08	1.87	1.98	1.89	2.32
Minimum	0.10	0.36	0.17	0.65	0.10	0.36
Maximum	5.58	7.07	4.77	7.07	5.58	6.51
Lower 95% CL mean	1.84	2.19	1.76	2.01	1.78	2.19
Upper 95% CL mean	2.17	2.63	2.24	2.64	2.23	2.80

Abbreviations: CF, cervical frame; CL, confidence level; C3, third cervical vertebra; SD, standard deviation.

The analysis of the SAA at the target point in the dorsoventral plane (y‐axis) revealed a mean ± SD SAA of 0.85 mm ± 0.67 mm in patient tracker position CF, and 1.15 ± 1.02 mm in patient tracker position C3 (Table S2). Y‐axis deviations exceeding 2 mm occurred in five of 72 measurements in the patient tracker position CF with the maximum value being 3.72 mm in a dorsal direction. In the patient tracker position C3, 12 of 72 measurements exceeded 2 mm, including one measurement exceeding 4 mm (5.2 mm in a ventral direction).

## 4 DISCUSSION

In this study, we tested a purpose‐built frame designed for stable fixation of the equine cervical vertebral column to facilitate CAS of the cervical vertebrae C3 to C5 via a ventral approach. When using the described equipment and set‐up for CAS, SAA remained within the close range of 2 mm, regardless of whether the patient tracker was positioned on the ventral crest of C3 or the lateral panel of the CF, aligning with our first hypothesis. Our second hypothesis of increased SAA in vertebrae more distant from the patient tracker could not be confirmed.

A positional accuracy with a mean error ≤2 mm is reported for the navigation system used in this study.^17^ Previous studies utilizing the same surgical navigation system and a purpose‐built frame to facilitate CAS in equine cadaveric limbs demonstrated surgical accuracy with a mean overall SAA of ≤1 mm and a maximum SAA of 3.4 mm.^16^, ^18^ SAAs in these studies were measured separately in individual anatomical planes rather than as Euclidean distances (the square root of the sum of squared values in three planes). As a result, slightly higher values are expected when using Euclidean distances, which account for all three dimensions simultaneously.

Several factors likely contributed to the SAAs in this study. (1) The substantial soft tissue mass around the cervical vertebrae makes rigid patient tracker fixation more challenging than in the equine distal extremity. (2) While the CF was designed to prevent twisting of the cervical vertebral column by allowing the crest of the neck to pass between the panels, this may also have permitted gradual neck displacement over time. (3) The neck and head weight could cause subtle plastic deformations of the CF, although no mechanical testing has been conducted to further evaluate this. (4) It was hard to avoid slipping of the drill bit on the steeply sloped ventral contour of the cervical vertebrae when starting free‐hand computer‐assisted drilling using a conventional battery‐powered surgical drill. To reduce inaccuracy arising from this phenomenon, we decided to use a navigated high‐speed drill, which has been demonstrated to reduce slipping at the entry point on oblique bone surfaces and improve drilling accuracy in combined robotic‐navigated surgery.^19^ Nonetheless, slipping of the drill likely remained a factor contributing to SAA. (5) To stabilize the cervical vertebrae, we adjusted the side panel spacing to wedge the transverse processes between the panels and inserted wedge‐shaped foam cushions to fill gaps. Despite this, a slight bouncing of the cervical vertebral column was observed when manually pushing down on the exposed vertebrae. However, a much greater force compared with the forces applied during computer‐assisted drilling had to be applied to produce this bouncing effect. The large ventral approach and extensive soft tissue dissection likely contributed to this phenomenon. In clinical settings, smaller approaches and less dissection are typical for single or double‐level fusions, depending on the implants used. While replacing foam cushions with custom hard plastic inserts could enhance stability, this must be weighed against an increased risk of myopathies in a clinical setting.

**FIGURE 6:**
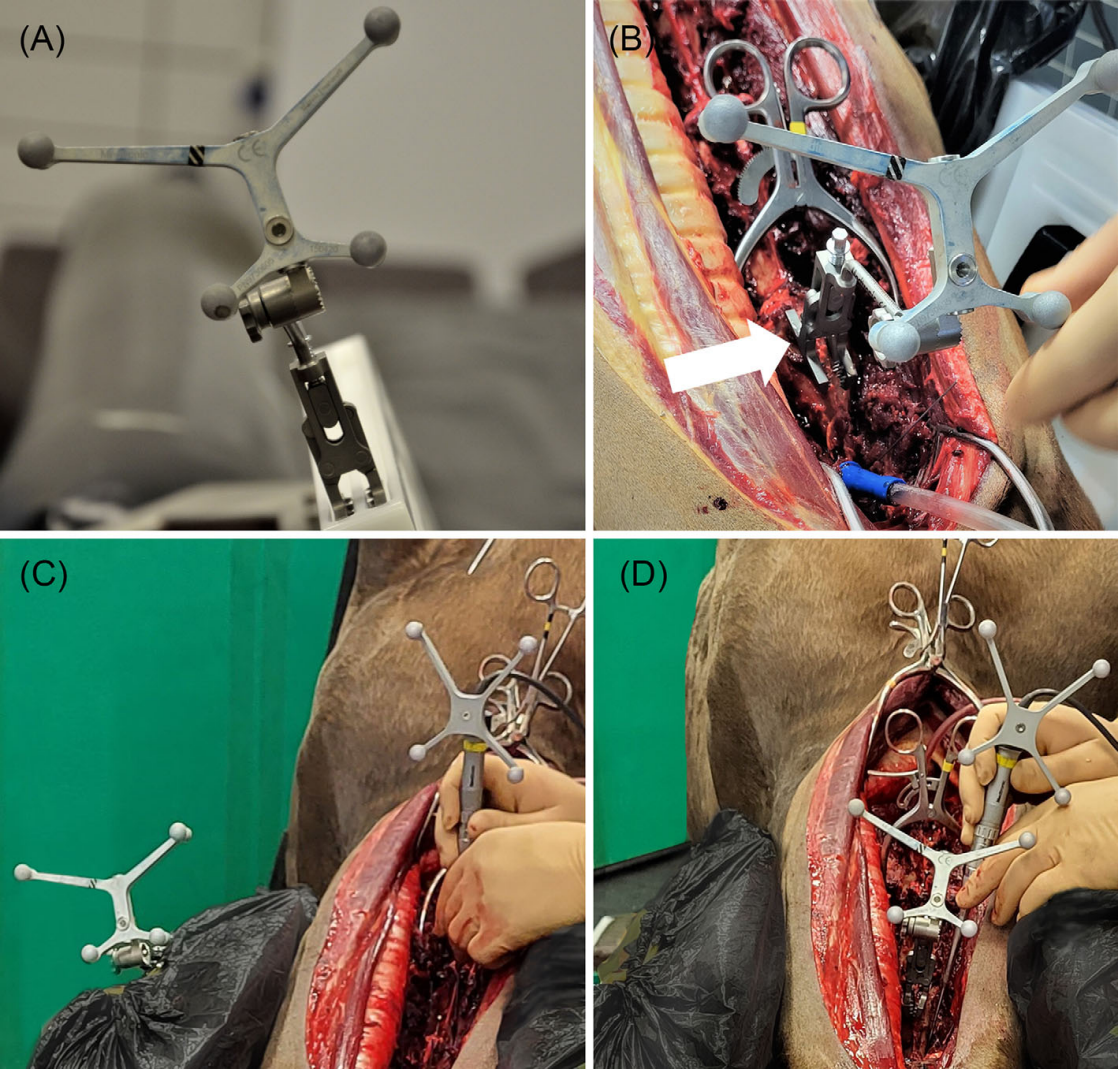
Demonstration of the two different patient tracker positions. (A) The patient tracker secured to the milled‐in recesses on the panel of the cervical frame using a spinous process clamp (Medtronic). (B) Patient tracker fixed to the ventral crest of C3 (white arrow) using the same spinous process clamp. (C, D) Intraoperative images showing the advantageous patient tracker positioning on the cervical frame (C), providing greater workspace and minimizing spatial interference compared to the restricted space when placed on the ventral crest of C3 (D).

Positioning the patient tracker in a strategically advantageous position on the CF effectively avoided spatial interference while at the same time not compromising surgical accuracy in this study (Figure 6C,D). Similar results have been shown using the purpose‐built frame for the equine distal extremity.^16^ In CAS with optical tracking, spatial interference can be caused by physical contact or superimposition between the instrument‐ and patient‐tracker or because their reflecting spheres are temporarily obscured from being detected by the localizer camera. Regardless of its cause, spatial interference can contribute to increased SAAs or prolong and complicate CAS procedures. Hence, the finding that the CF stabilizes the vertebral column sufficiently to allow for the positioning of the patient tracker outside the surgical field and without compromising surgical accuracy is critically relevant. Besides spatial interference, an unstable fixation or accidental displacement of the patient tracker can also significantly compromise surgical accuracy and prolong CAS procedures.^20^ Fixing the same spinous process clamp and patient tracker to the milled‐in depressions of a panel rather than to the ventral crest of C3 reduced the risk of accidental displacement and provided a more stable attachment (Figure 6A,B).

The statistical analyses revealed no evidence that the vertebral level (C3 to C5) influenced the achieved mean SAA (*p* = .1), refuting our second hypothesis. In human posterior spinal neuronavigation procedures, attaching a patient tracker to the spinous process of a single selected vertebra in the surgical field is common practice.^21^, ^22^ In this case, the spine needs to be immobilized by applying a (Mayfield) skull clamp, by stabilizing the spinal column with a pillow, or by using specialized tables for spine surgeries.^21^, ^22^ Nevertheless, it was reported that the incidence of malpositioned pedicle screws increases significantly in spinal segments three levels or more away from the patient tracker in human scoliosis surgery. It has been speculated that this is attributed to intervertebral movements caused by the surgical manipulation.^14^, ^22^ Our results indicate that this effect did not occur in our study, further supporting the CF's ability to maintain stability for CAS across multiple vertebral segments in the equine cervical vertebral column. However, the lack of evidence regarding an influence of vertebral level on the SAA may be due to insufficient statistical power.

In clinical practice, excessive surgical deviation toward the spinal canal could lead to spinal cord injury. Based on our experiences and the results of this study, we consider the here‐described setup as safe for computer‐assisted drilling in a clinical context of vertebral interbody fusion surgery in horses, with the vast majority of recorded dorsoventral deviations at the drill corridor target point being <2 mm. In pedicle screw fixations in humans, a margin of error of 2 mm (medial pedicle perforation) is considered safe by most surgeons.^14^, ^15^ Considering the anatomical proportions of the equine vertebrae, accepting a safety margin of this magnitude appears reasonable. Nonetheless, outliers should be accounted for during all stages of CAS, including surgical planning and computer‐assisted drilling. In this context, it is crucial to regularly verify setup accuracy by matching an identifiable anatomical landmark on the patient with its corresponding image on the screen,^19^, ^23^ as was done in our experiments by verifying the virtual position of the spherical head screw.

Before clinical application of the CF, potential sources of patient morbidities associated with the current design of the CF need to be considered and addressed. As the CF is designed from hard plastic polymer, it is important to provide sufficient padding to avoid myopathies of the neck, shoulder, and withers. Furthermore, prolonged hyperextension of the neck has been associated with recurrent laryngeal nerve damage and dysfunction.^5^ To reduce this risk, an appropriate head support that suspends the head in a less extended position has meanwhile been integrated in the CF.

This study has several limitations. All but one cadaver developed noticeable stiffness of the neck musculature caused by the onset of rigor mortis at the end of the experiment. This increasing stiffness could potentially have influenced the stability of the setup and, consequently, SAA over time. However, the analysis of the chronology of computer‐assisted drilling, reflecting whether drill corridors were prepared during the first or second patient tracker position, showed no evidence of an effect of neck stiffness on SAA. In live animals, the potential impact of respiratory motion on surgical accuracy should be considered. Our investigations were focused on cervical vertebral levels C3 to C5. Extrapolations to other cervical vertebral regions using the same surgical navigation system and CF are limited. Similarly, only horses weighing 400 to 600 kg were included. Appropriate patient positioning and feasibility of the navigated procedure using the same CF in larger horses and at more caudal locations (i.e., C6–T1) remain to be evaluated. Importantly, we did not perform mechanical testing of the CF. Although it supported the weight of the neck in cadavers weighing up to 600 kg, standardized mechanical testing to establish its safety range and fully approve its clinical applications is advised.

## 5 CONCLUSION

The CF was useful for CAS of the equine cervical vertebral column via a standard ventral approach in a cadaveric model. It allowed for unrestricted pre‐ and intraoperative CBCT imaging, computer‐assisted drilling with SAA in the close range of 2 mm, and patient tracker positioning in a location remote from the restricted surgical field. Surgical accuracy was preserved at one and two vertebral levels distant from the patient tracker. Future studies should focus on the mechanical properties of the CF, its suitability for application at more caudal cervical vertebral levels, and practical considerations for clinical use, such as further refinements to prevent patient morbidity.

## AUTHOR CONTRIBUTIONS

All authors have contributed to this project and the resulting publication. Maurer T, Dr Med Vet: Study design, development of the cervical frame, execution of the experiments, data acquisition, data analysis, and manuscript preparation. de Preux M, Dr Med Vet, DECVS: Study design, development of the cervical frame, data analysis, and manuscript preparation. Precht C, Dr Med Vet, DECVDI, PhD: Study design, imaging, and manuscript preparation. Vidondo B, MScBiol, MSc, IT, PhD: Data analysis and manuscript preparation. Koch C, Dr Med Vet, DACVS (Large Animal), DECVS: Initiation of the project, study design, development of the cervical frame, execution of the experiments, data acquisition, and manuscript preparation.

## FUNDING INFORMATION

This study was supported by a Resident Research Grant from the European College of Veterinary Surgeons (ECVS) and a research grant from the Specialization Commission of the Vetsuisse Faculty, University of Bern.

## CONFLICT OF INTEREST STATEMENT

The authors declare no conflict of interest related to this report.

## Supporting information


**Table S1.** Distribution of surgical accuracy aberration (SAA) in mm (Euclidean distance) on each vertebra.

**Table S2.** Distribution of surgical accuracy aberration (SAA) measurements in mm in mediolateral direction (*x*‐axis), ventrodorsal direction (y‐axis), and caudocranial direction (*z*‐axis).
