# Supplementary material for: Accuracy of computer‐assisted drilling of equine cervical vertebral bodies using a purpose‐built cervical frame—An experimental cadaveric study
Source: Vet Surg. 2025 May 15;54(5):898–908. doi: 10.1111/vsu.14271 (PMC12282039; doi:10.1111/vsu.14271)
Supplement: Supplementary file 1 — Table S1. Distribution of surgical accuracy aberration (SAA) in mm (Euclidean distance) on each vertebra. [file VSU-54-898-s001.docx]

**Table S1:**

Distribution of surgical accuracy aberration (SAA) in mm (Euclidean distance) on each vertebra

| SAA | Vertebra C3 | | | Vertebra C4 | | | Vertebra C5 | | |
| --- | --- | --- | --- | --- | --- | --- | --- | --- | --- |
| Patient tracker position | CF | C3 | **Combined**  **(all measures)** | CF | C3 | **Combined (all measures)** | CF | C3 | **Combined (all measures)** |
| Mean | 1.88 | 2.01 | **1.94** | 1.78 | 2.56 | **2.17** | 2.36 | 2.67 | **2.51** |
| SD | 0.88 | 1.18 | **1.04** | 0.79 | 1.28 | **1.13** | 1.16 | 1.39 | **1.28** |
| Median | 1.85 | 1.87 | **1.86** | 1.77 | 2.43 | **2.01** | 2.31 | 2.23 | **2.31** |
| Minimum | 0.10 | 0.50 | **0.10** | 0.49 | 0.36 | **0.36** | 0.17 | 0.84 | **0.17** |
| Maximum | 3.97 | 7.07 | **7.07** | 3.65 | 5.99 | **5.99** | 5.58 | 6.51 | **6.51** |
| Lower 95% CL mean | 1.62 | 1.66 | **1.73** | 1.55 | 2.18 | **1.94** | 2.02 | 2.26 | **2.25** |
| Upper 95% CL mean | 2.13 | 2.35 | **2.15** | 2.01 | 2.93 | **2.40** | 2.69 | 3.07 | **2.77** |

Abbreviations: CF, cervical frame; CL, confidence level; C3, third cervical vertebra; SD, standard deviation
