# Supplementary material for: Accuracy of computer‐assisted drilling of equine cervical vertebral bodies using a purpose‐built cervical frame—An experimental cadaveric study
Source: Vet Surg. 2025 May 15;54(5):898–908. doi: 10.1111/vsu.14271 (PMC12282039; doi:10.1111/vsu.14271)
Supplement: Supplementary file 2 — Table S2. Distribution of surgical accuracy aberration (SAA) measurements in mm in mediolateral direction (x‐axis), ventrodorsal direction (y‐axis), and caudocranial direction (z‐axis). [file VSU-54-898-s002.docx]

| SAA | | Entry | | Target | |
| --- | --- | --- | --- | --- | --- |
| Patient tracker position | | CF | C3 | CF | C3 |
| Mean | x | 0.60 | 0.93 | 0.96 | 1.11 |
|  | y | 1.31 | 1.24 | 0.85 | 1.15 |
|  | z | 1.10 | 1.39 | 1.30 | 1.55 |
| SD | x | 0.44 | 0.64 | 0.64 | 0.77 |
|  | y | 0.95 | 1.16 | 0.67 | 1.02 |
|  | z | 0.82 | 1.05 | 0.87 | 1.16 |
| Lower 95% CL mean | x | 0.50 | 0.78 | 0.81 | 0.93 |
|  | y | 1.08 | 0.96 | 0.70 | 0.91 |
|  | z | 0.91 | 1.15 | 1.09 | 1.28 |
| Upper 95% CL mean | x | 0.71 | 1.08 | 1.11 | 1.30 |
|  | y | 1.53 | 1.51 | 1.01 | 1.39 |
|  | z | 1.29 | 1.64 | 1.50 | 1.82 |
| Median | x | 0.53 | 0.81 | 0.93 | 1.08 |
|  | y | 1.14 | 0.89 | 0.68 | 0.81 |
|  | z | 1.03 | 1.26 | 1.10 | 1.17 |
| Minimum | x | 0.01 | 0.08 | 0.01 | 0.03 |
|  | y | 0.09 | 0.04 | 0.01 | 0.06 |
|  | z | 0.00 | 0.07 | 0.04 | 0.04 |
| Maximum | x | 1.91 | 2.53 | 2.78 | 2.82 |
|  | y | 4.26 | 5.69 | 3.72 | 5.20 |
|  | z | 3.39 | 4.76 | 3.38 | 4.71 |
| Medial:lateral | x | 48:24 | 52:20 | 39:33 | 29:43 |
| Ventral:dorsal | y | 53:19 | 47:25 | 50:22 | 32:40 |
| Caudal:cranial | z | 33:39 | 16:56 | 44:28 | 22:50 |

**Table S2:**

Distribution of surgical accuracy aberration (SAA) measurements in mm in mediolateral direction (x-axis), ventrodorsal direction (y-axis), and caudocranial direction (z-axis):

Abbreviations: CF, cervical frame; CL, confidence level; C3, third cervical vertebra; SD, standard deviation
